# Styrylpyridinium Derivatives as New Potent Antifungal Drugs and Fluorescence Probes

**DOI:** 10.3389/fmicb.2020.02077

**Published:** 2020-08-28

**Authors:** Simona Vaitkienė, Rimantas Daugelavičius, Hana Sychrová, Marie Kodedová

**Affiliations:** ^1^Department of Biochemistry, Faculty of Natural Sciences, Vytautas Magnus University, Kaunas, Lithuania; ^2^Laboratory of Membrane Transport, Division Biotechnology and Biomedicine Centre of the Academy of Sciences and Charles University (BIOCEV), Institute of Physiology of the Czech Academy of Sciences, Vestec, Czechia

**Keywords:** styrylpyridinium derivatives, *Candida glabrata*, diS-C_3_(3) assay, membrane potential, yeast, multidrug resistance, vacuolar marker

## Abstract

The incidence of *Candida glabrata* infections increases every year due to its higher resistance to commonly used antifungal drugs. We characterized the antifungal mechanism of action of eight new styrylpyridinium derivatives, with various N-alkyl chains (-C_6_H_13_, -C_8_H_17_, -C_10_H_21_, -C_12_H_25_) and different substituents, on *C. glabrata* strains differing in their drug resistance due to the presence or absence of two major drug-efflux pumps. We found that the tested styrylpyridinium compounds affected the growth of *C. glabrata* cells in a compound- and strain-dependent manner, and apparently they were substrates of *Cg*Cdr1 and *Cg*Cdr2 pumps. Further, we determined the impact of the tested compounds on plasma membrane integrity. The ability to cause damage to a plasma membrane depended on the compound, its concentration and the presence of efflux pumps, and corresponded well with the results of growth and survival tests. We also tested possible synergism with three types of known antifungal drugs. Though we did not observe any synergism with azole drugs, styrylpyridinium compounds **5** and **6** together with FK506 demonstrated excellent antifungal properties, whereas compounds **2**, **3**, **5**, and **6** exhibited a significant synergistic effect in combination with terbinafine. Based on our results, derivatives **2** and **6** turned out to be the most promising antifungal drugs. Moreover, compound **6** was not only able to effectively permeabilize the yeast plasma membrane, but also exhibited significant synergism with FK506 and terbinafine. Finally, we also characterized the spectroscopic properties of the tested styrylpyridinium compounds. We measured their absorption and fluorescence spectra, determined their localization in yeast cells and found that their fluorescence characteristics differ from the properties of current commercial vacuolar styrylpyridinium markers and allow multi-color staining. Compounds **1**, **3**, **7**, and **8** were able to accumulate in plasma and vacuolar membranes, and compounds **2**, **5**, and **6** stained the whole interior of dead cells. In summary, of the eight tested compounds, compound **6** is the most promising antifungal drug, compound **8**, due to its minimal toxicity, is the best candidate for a new vacuolar-membrane probe or new benchmark substrate of *C. glabrata* Cdr pumps, and derivative **5** for a new vital dye.

## Introduction

Infections caused by fungal pathogens have become a critical health problem, especially for immunocompromised individuals. Even though *Candida albicans* persist as the most common species causing invasive candidiasis, the number of non-*albicans* (particularly *C. glabrata*) infections is intensively increasing worldwide every year. The rising incidence of *C. glabrata* infections occurs as a result of the higher resistance of *C. glabrata* to widely used antifungal drugs, such as azoles, polyenes, echinocandins, and flucytosine (Ksiezopolska and Gabaldón, 2018). The majority of current antifungal drugs target ergosterol (polyenes) and its biosynthetic pathway (azoles, allylamines, and morpholines). Azoles, but also allylamines, such as terbinafine, disturb the function of the yeast plasma membrane by inhibiting various enzymes in the ergosterol biosynthetic pathway, resulting in the depletion of ergosterol together with the accumulation of toxic sterols in the membrane (Petrikkos and Skiada, 2007). Nevertheless, the fungistatic action of azoles often evokes the development of resistance, for example *C. glabrata* exhibits higher innate and acquired resistance to the azole class of antifungals than *C. albicans* (Pais et al., 2019). This resistance is often associated with the overexpression of two multidrug resistance (MDR) transporters – *Cg*Cdr1 and *Cg*Cdr2 (Cannon et al., 2009).

Increase in multidrug resistance is a serious problem, and a novel treatment for fungal infections is urgently needed. Finding new antifungal molecules could deal with the rising prevalence of *C. glabrata*. Promising drug candidates can be also searched for among compounds that are currently used for very different applications, e.g., the painkiller/anti-inflammatory drug diclofenac potentiates the activity of caspofungin, while it attenuates the effectiveness of fluconazole against *C. albicans* (Mathé and Van Dijck, 2013; Urai et al., 2014). Likewise, lipophilic styrylpyridinium dyes (FM4-64 and FM1-43) were used for a long time as efficient fluorescence probes for staining and analyzing cell membrane features and pathologies (Heese-Peck et al., 2002; Bolte et al., 2004; Miner et al., 2019). Only a few studies demonstrated that some styrylpyridinium compounds may also have antibacterial effects (Wyrzykiewicz et al., 1994; Chanawanno et al., 2010). Therefore we focused on determining and comprehensively analyzing the antifungal mechanism of action of styrylpyridinium derivatives to evaluate their possible application in the treatment of fungal infections. In our previous study (Vaitkienė et al., 2020), we newly synthetized six styrylpyridinium compounds and, together with seven already known styrylpyridinium derivatives, investigated their fungicidal effect, synergism with fluconazole, and cytotoxicity to mammalian cells. Our results indicated that eight compounds were able to reduce *C. albicans* growth and some of them were substrates of *C. albicans* MDR pumps, since different concentrations were required to inhibit the growth of *C. albicans* strains with deleted *CDR1*, *CDR2*, or *MDR1* genes (Vaitkienė et al., 2020).

The aim of this study was to characterize the mechanism of action of eight styrylpyridinium compounds, which were selected based on their highest efficiency against *C. albicans* in our previous study (Vaitkienė et al., 2020), on *C. glabrata* strains differing in their ability to export drugs. We used the potentiometric fluorescence probe diS-C_3_(3) to evaluate the impact of these drugs on plasma membrane integrity and identify *Cg*Cdr1 substrates, since the diS-C_3_(3) probe not only accumulates in yeast cells in response to membrane potential (Gášková et al., 1998), but is also a substrate of *Cg*Cdr1 (Kodedová and Sychrová, 2016), thus helping to identify other substrates of this drug-efflux pump via competitive inhibition among its substrates. We also monitored *C. glabrata* growth in the presence of styrylpyridinium derivatives and tested possible synergism with known antifungal drugs and substrates of *Cg*Cdr1 and/or *Cg*Cdr2 pumps. Further, we performed a basic spectroscopic analysis of these compounds and found possible applications of the styrylpyridinium derivatives with low toxicity as new fluorescence probes in yeast research.

## Materials and Methods

### Styrylpyridinium Derivatives

**FIGURE 1:**
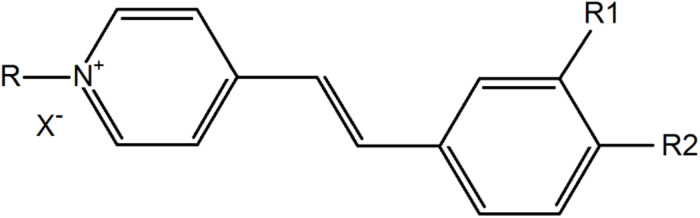
Basic structure of styrylpyridinium derivatives.

Styrylpyridinium derivatives were synthesized according to a well-known two-step procedure involving the alkylation of γ-picoline with an appropriate alkyl bromide and further condensation with substituted benzaldehyde (Vaitkienė et al., 2020). Figure 1 illustrates the basic structure of styrylpyridinium derivatives, the particular substituents of these 8 compounds used in this study are summarized in Table 1. The compounds were dissolved in ethanol, usually as 2 mg/mL stock solutions.

**TABLE 1:** Styrylpyridinium derivatives used in this study.

**Compound**	**R1**	**R2**	**R**	**X^–^**	**References**
**1**	-H	CH_3_-CH-N-CH-CH_3_	-C_12_H_25_	Br**^–^**	Vaitkienė et al., 2020
					
**2**	-H	-OH	-C_12_H_25_	Br**^–^**	Wyrzykiewicz et al., 1994
**3**	-O-CH_3_	-O-CH_3_	-C_12_H_25_	Br**^–^**	Vaitkienė et al., 2020
**4**	-H	-CN	-C_12_H_25_	Br**^–^**	Vaitkienė et al., 2020
**5**	-H	-OH	-C_8_H_17_	Br**^–^**	Krieg et al., 2007
**6**	-H	-OH	-C_10_H_21_	Br**^–^**	Mishra et al., 2001
**7**	-H	CH_3_-N-CH_3_	-C_8_H_17_	Br**^–^**	Shiraishi et al., 2010
					
**8**	-H	CH_3_-N-CH_3_	-C_6_H_13_	CH_3_-C_6_H_4_SO_3_^–^	Dubur et al., 1984
					

### Yeast Strains and Growth Conditions

*Candida glabrata* strains used in this study are listed in Table 2. Cells were grown at 30°C in YPD medium (1% yeast extract, 2% peptone, 2% glucose, and 2% agar for solid media). For experiments, cells from the early exponential growth phase were harvested, washed twice with sterile deionized water and resuspended in deionized water (for fluorescence microscopy) or in 10 mM citrate-phosphate (CP) buffer (pH 6.0) to OD_600_ = 0.2.

**TABLE 2:** Yeast strains used in this study.

**Strain**	**Genotype or phenotype**	**References**
DSY565	*C. glabrata* clinical isolate, resistant to azoles	Sanglard et al., 1999
DSY1041	*cdr1*Δ*::hisG-URA3-hisG*	Sanglard et al., 1999
DSY1612	*cdr2*Δ*::hisG-URA3-hisG*	Sanglard et al., 2001
DSY1613	*cdr2*Δ*::hisG cdr1*Δ*::hisG-URA3-hisG*	Sanglard et al., 2001

### Determination of Plasma-Membrane Integrity [diS-C_3_(3) Assay]

The relative membrane potential and integrity of the yeast cell plasma membrane was estimated by a fluorescence assay based on the potential-dependent redistribution of the fluorescence probe diS-C_3_(3) (3,3′-dipropylthiacarbocyanine iodide) (Denksteinová et al., 1997; Gášková et al., 1998), as described in Kodedová et al. (2019). The probe was added to a final concentration of 0.02 μM to yeast suspensions in CP buffer, and samples were occasionally gently stirred. Fluorescence emission spectra (λ_ex_ = 531 nm, λ_em_ = 560–590 nm) of the cell suspensions were measured in a FluoroMax-4 spectrofluorimeter (Horiba Scientific). The staining curves represented the dependence of the fluorescence emission maximum wavelength λ_max_ on the time of staining. The tested compounds were usually added after ∼ 10 min of staining. Representative results of at least three independent experiments (always with similar results) are shown.

### Estimation of Cell Survival (Plating Test)

To determine cell viability, *C. glabrata* cells resuspended in CP buffer were treated with the styrylpyridinium derivatives with occasional gentle stirring for 20 min. 10 μL of cell suspensions were then diluted 100-fold, and aliquots were spread on YPD agar plates in triplicates. The number of colonies (CFU) was determined after 1 day of incubation of the plates at 30°C. Experiments were repeated three times. Results are shown in the form of percentages as means ± SD; the control sample without exposure to the drugs was regarded as 100% cell survival.

### Growth Curves

To compare the resistance of strains to styrylpyridinium derivatives, the growth in liquid medium was monitored in a 96-well plate reader Elx808 (BioTek) with shaking at 30°C for 24 h. Cells were cultivated in 100 μL of YPD medium inoculated to OD_600_ = 0.001. The OD_600_ was measured at 1 h intervals. Growth curves were obtained in duplicates over a broad range of drug concentrations (0.5–20 μM). The plotted values of relative growth after 24 h cultivation are the means ± SD of three independent experiments.

### Disk Diffusion Tests

Disk diffusion tests were performed for estimating the synergistic effect of styrylpyridinium derivatives with FK506, terbinafine, fluconazole and ketoconazole. Washed yeast cells were diluted into YPD top agar (1% agar) to OD_600_ = 0.2 and poured onto solidified YPD plates (2% agar). Drug solutions (2 μL) at the concentrations indicated in the text were spotted onto paper disks laid on top of the solidified agar with cells. The plates were photographed after 1 day of incubation at 30°C. Representative results of three independent experiments are shown.

### Measurement of Absorption and Fluorescence Spectra of Styrylpyridinium Derivatives

The absorption spectra of 20 μM styrylpyridinium derivatives in deionized water were measured in a quartz cuvette with an optical path length of 10 mm using an Agilent 8453 UV-visible spectrophotometer in the range 190–1100 nm. All spectra were corrected for the baseline. Maximum absorption wavelengths (λ_abs_) summarized in Table 3 were used for the fluorescence excitation of the given compounds. The fluorescence emission spectra of 20 μM styrylpyridinium derivatives in deionized water were measured in a quartz cuvette with a FluoroMax-4 spectrofluorimeter in the range 350–1000 nm. Fluorescence spectra were normalized to the unified maximum fluorescence intensity. The Stokes shift (Table 3) was calculated as the difference between the spectral position of the maximum of the absorption (λ_abs_) and the maximum of the fluorescence emission (λ_em_) expressed in wavelength units.

**TABLE 3:** Maximum absorption and fluorescence emission wavelengths and corresponding Stokes shifts of styrylpyridinium derivatives.

**Compound**	**λ_abs_ (nm)**	**λ_em_ (nm)**	**Stokes shift (nm)**
**1**	474	609	135
**2**	377	505	128
**3**	384	531	147
**4**	335	405	70
**5**	375	505	130
**6**	375	504	129
**7**	450	603	153
**8**	452	605	153

### Fluorescence Microscopy

Washed *C. glabrata* DSY565 cells were resuspended in deionized water, stained with 2 μM styrylpyridinium derivatives and after cca 10 min observed with a Leica TCS SP8 WLL SMD confocal fluorescence microscope (objective HC PL APO CS2 63×/NA 1.20 with water immersion, λ_ex_ = 405 nm, green fluorescence λ_em_ = 480–650 nm, red fluorescence λ_em_ = 650–800 nm). For the demonstration of the different accumulation of styrylpyridinium derivatives in yeast cells with damaged and intact plasma membranes, *C. glabrata* cells were resuspended in deionized water to OD_600_ = 0.2 and treated with 3 μM octenidine dihydrochloride (ODDC), causing full permeabilization of yeast cells within 15 min (Kodedová et al., 2019). Then the cells were washed with deionized water and used as a model of killed cells for styrylpyridinium staining.

### Styrylpyridinium Efflux Assay

The styrylpyridinium compound **8** was added to a final concentration of 2 μM to yeast suspensions (OD_600_ = 0.2) in CP buffer, and samples were occasionally gently stirred. Fluorescence emission spectra (λ_ex_ = 452 nm, λ_em_ = 500–750 nm) of the cell suspensions were measured in a FluoroMax-4 spectrofluorimeter (Horiba Scientific). The excitation and emission slits were set to 4 nm. The staining curves represented the time-dependence of the fluorescence intensity registered at emission maximum wavelength. The results are shown as means ± SD of three independent experiments.

### Statistical Analysis

The statistical analyses were performed with SigmaPlot 13. ANOVA with subsequent *post-hoc* test was used for identifying significant differences.

## Results and Discussion

### Impact of Styrylpyridinium Derivatives on Growth of *C. glabrata* Strains Differing in the Number of Their Drug-Efflux Pumps

**FIGURE 2:**
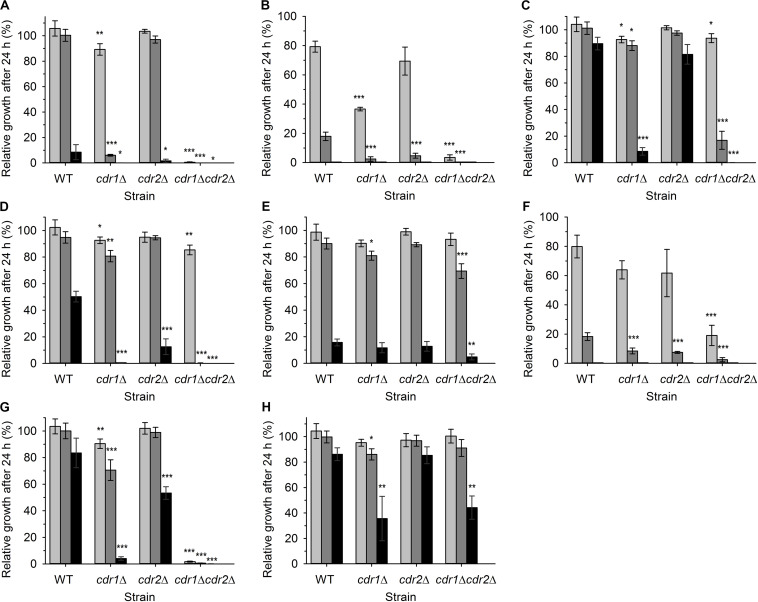
Relative growth of four *C. glabrata* strains in liquid YPD medium supplemented with various concentrations of styrylpyridinium derivatives **1(A)**, **2(B)**, **3 (C)**, **4 (D)**, **5 (E)**, **6 (F)**, **7 (G)**, and **8 (H)** in 24 h. Growth in YPD medium without any drugs was regarded as 100%. **(A–G)** 0.5 μM drugs (light gray bars), 1 μM drugs (dark gray bars), 2 μM drugs (black bars), **(H)** 5 μM compound **8** (light gray bars), 10 μM compound **8** (dark gray bars), 20 μM compound **8** (black bars). The *P*-values (*P < 0.05, **P < 0.01, ***P < 0.001) denote statistically significant differences from the wild-type strain.

First, we assessed the effect of compound addition on the growth of yeast cultures. We used a wild-type azole-resistant clinical isolate DSY565 and its three mutants lacking one or two MDR pumps, DSY1041 (*cdr1*Δ), DSY1612 (*cdr2*Δ), and DSY1613 (*cdr1*Δ *cdr2*Δ). The high antifungal resistance of DSY565 strain is caused by significant upregulation of *CDR1*, while *CDR2* is only moderately expressed (at the basal level) in this strain (Sanglard et al., 2001). Comparison of the growth inhibition among the four strains may suggest whether the used compounds are substrates of the two MDR pumps or not. We monitored the growth in YPD cultures in the presence of three different concentrations (0.5, 1, and 2 μM) of compounds **1**–**8** (Figure 1 and Table 1) for 24 h. The obtained results are summarized in Figure 2. All the tested compounds exhibited at least some inhibitory effect at the used range of concentrations except for compound **8**, for which concentrations an order of magnitude higher (5, 10, and 20 μM) were required to obtain any significant inhibition of *C. glabrata* growth (Figure 2H). The growth of wild-type cells was the most inhibited by compounds **2** and **6,** already at 1 μM concentration (Figures 2B,F), a little bit less by compounds **1**, **4**, and **5** (significant inhibition visible at 2 μM concentration, Figures 2A,D,E), and the presence of compounds **3** and **7** caused only a very slight decrease in growth when these two compounds were present at a concentration of 2 μM (Figures 2C,G).

The inhibitory effect of almost all the tested compounds was more pronounced in the cultures of mutant cells. The only exception was compound **5**, whose effects were quite similar for all 4 strains (Figure 2E). Cells lacking Cdr1 were more sensitive to all the tested compounds than the wild type, suggesting that they are probably effectively pumped by Cdr1 in the wild-type cells. The absence of Cdr1 was the most pronounced with compounds **2** and **6** (Figures 2B,F). The obtained results suggested that Cdr2 may also export the majority of the tested compounds, as the absence of both pumps in the double mutant led to the highest sensitivity of cells to all the tested compounds except compound **8** (Figure 2).

In summary, compounds **2** and **6** were the most toxic for wild-type cells and their toxicity was tremendously increased in the absence of Cdr pumps. Compound **8** was not significantly toxic to *C. glabrata* cells and compound **5**, though toxic at the 2 μM concentration, is probably not a good substrate of Cdr1 and Cdr2. When we compared the growth curves (not shown), we realized that styrylpyridinium derivatives affected their shape in a similar way, they predominantly prolonged the lag phase of growth curves (in the range of several hours) rather than slowing down the rate of exponential growth. Doubling times of wild-type and mutant strains cultivated in the presence of 0.5 μM styrylpyridinium compounds differed on average of 5 min, except more toxic compounds **2** and **6,** which prolonged doubling times of some strains to 11 and 22 min (doubling times of *cdr1*Δ *cdr2*Δ strain were not determined in the presence of compounds **1**, **2**, **6**, and **7**, as cell cultures did not reach exponential growth phase during 24-h cultivation). The prolongation of lag phase could be explained by a transitional period necessary for cell adaptation to the presence of drugs (induction of expression of Cdr pumps) or by the immediate killing of a certain amount of inoculated cells upon their primary contact with styrylpyridinium compounds. Of course, both of the above may occur together. To elucidate whether the used compounds were killing cells rapidly, we investigated the changes in plasma-membrane integrity upon the addition of styrylpyridinium derivatives by the diS-C_3_(3) fluorescence assay.

### Effect of Styrylpyridinium Derivatives on the Cell Plasma-Membrane Integrity

The diS-C_3_(3) assay monitors the rate of influx of this cationic probe into cells, which is driven by the internal negative membrane potential in intact cells. A very rapid increase in staining suggests that the integrity of plasma membrane is disrupted and the probe enters cells independently of the membrane potential (Kodedová et al., 2011, 2019). Moreover, comparing the changes in staining curves of wild-type and *cdr1*Δ cells upon the addition of the tested compounds should confirm the results of the growth test, i.e., the ability of Cdr1 to eliminate the tested styrylpyridinium compounds from cells.

**FIGURE 3:**
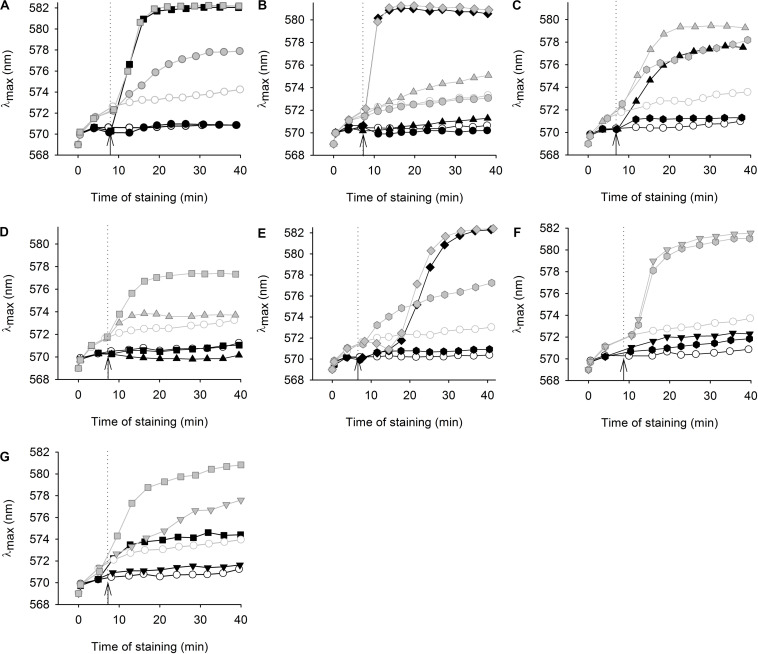
Effect of styrylpyridinium derivatives on the staining of two *C. glabrata* strains with a potentiometric probe. Changes in diS-C_3_(3) staining of wild-type (black symbols) and *cdr1*Δ (gray symbols) cells after addition of styrylpyridinium derivatives (full symbols); **(A)** 1 μM (circles) and 10 μM (squares) compound **2**, **(B)** 1 μM (circles), 5 μM (triangles) and 20 μM (diamonds) compound **3**, **(C)** 0.5 μM (hexagons) and 5 μM (triangles) compound **4**, **(D)** 5 μM (triangles) and 10 μM (squares) compound **5**, **(E)** 0.5 μM (hexagons) and 20 μM (diamonds) compound **6**, **(F)** 0.5 μM (hexagons) and 2 μM (inverted triangles) compound **7**, **(G)** 2 μM (inverted triangles) and 10 μM (squares) compound **8**. Staining of control cells without styrylpyridine addition (empty circles); arrows with dotted lines indicate the addition of compounds.

**FIGURE 4:**
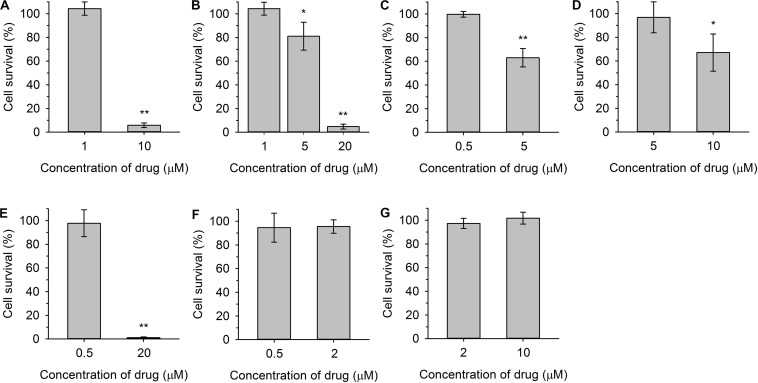
Viability of *cdr1*Δ cells after 20-min exposure to indicated concentrations of styrylpyridinium compounds **2(A)**, **3(B)**, **4 (C)**, **5 (D)**, **6 (E)**, **7 (F)**, and **8 (G)**. The control sample without exposure to the drugs was regarded as 100% cell survival. The *P*-values (*P < 0.05, **P < 0.001) denote statistically significant differences compared to the control.

We tested the effects of styrylpyridines (over a broad range of concentrations from 0.5–20 μM) on the wild type and *cdr1*Δ mutant. In this screening, we had to leave out compound **1**, because it strongly interacted with the diS-C_3_(3) probe and disturbed its fluorescence emission spectrum (not shown). The most interesting results obtained for compounds **2**–**8**, illustrating the effect of styrylpyridinium derivatives on plasma-membrane integrity and the Cdr1-mediated efflux of these compounds, are summarized in Figure 3. Simultaneously, to distinguish between minor damage or only harmless hyperpolarization/higher staining caused by styrylpyridines and serious damage leading to the membrane permeabilization, *cdr1*Δ cell viability was estimated after 20-min exposure to the tested compounds (Figure 4).

Figure 3A shows an example of a severe plasma-membrane damage caused by 10 μM compound **2**, as well as confirming it to be a substrate of Cdr1. Control curves (empty circles) showed a higher staining of the *cdr1*Δ mutant (gray) than of the wild-type cells (black). The difference corresponds to a higher amount of the probe in *cdr1*Δ cells. Compound **2** was confirmed to be a substrate of Cdr1, since its presence at a relatively low concentration (1 μM; full circles) led to a higher staining of *cdr1*Δ cells (Figure 3A, full gray circles), but not of wild-type cells possessing Cdr1. Brief exposure to a 1 μM concentration of compound **2** did not kill *cdr1*Δ cells (Figure 4A). On the other hand, the addition of 10 μM compound **2** (full squares) caused an immediate and huge increase in staining, which corresponds to a permeabilization of the membranes of both strains (Figure 3A). This rapid permeabilization resulting in the loss of viability was confirmed in the plating test (Figure 4A).

As with compound **2**, we confirmed the ability of Cdr1 to also mediate the efflux of the other styrylpyridinium derivatives from *C. glabrata* cells (Figure 3). Differences in the effects of the individual compounds on the staining curves suggest that Cdr1 exports these compounds with different affinities. Some of the compounds, e.g., **3** and **4**, significantly affected the membrane integrity at a concentration as low as 5 μM, and severe membrane damage was observed when the concentration of compounds was 10 μM or higher (Figures 3, 4). As in the growth tests, compound **8** had the lowest negative effect of all the tested compounds. Though the presence of a 10 μM concentration caused a clearly pronounced increase in the staining curve (Figure 3G), it was not accompanied by a loss of viability (Figure 4G). Also compound **7**, possessing a longer alkyl chain R than derivative **8**, was able to cause a non-lethal massive increase in staining of *cdr1*Δ cells, in fact the highest shift in staining at the lowest concentration (0.5 μM) of all the tested drugs (Figure 3F).

Compounds **2** and **6** were the best inhibitors of growth (Figures 2B,F) and were also highly potent at causing rapid membrane damage leading to the loss of cell viability (Figures 3A,E, 4A,E). 10 μM compound **2** and 20 μM compound **6** completely permeabilized plasma membranes of both strains independently on the presence of Cdr1. In these cases, Cdr1 pump is no longer able to protect the cells from drugs. Nevertheless, the permeabilization of the plasma membrane with compound **6** occurred later than that caused by compound **2** (Figures 3A,E), which suggested a difference in their mode of action, and might be connected to their molecular structure. Compound **6** has a shorter hydrophobic aliphatic R substituent than compound **2** (Table 1). Compared to compounds **2** and **6**, the third derivative with a hydroxyl substituent and the shortest alkyl chain (R), compound **5**, produced a lower effect at the 5 μM concentration (Figure 3D). In summary, this derivative was the least toxic of all the hydroxystyrylpyridines in both the short and long term (Figures 2E, 3D, 4D).

In summary, we demonstrated that the effects of styrylpyridinium derivatives on the plasma membrane integrity of *C. glabrata* cells were dependent on their structure and concentrations. Longer aliphatic R chains (Table 1) in groups of derivatives that were otherwise the same caused a stronger impairment of the plasma membrane. Analogously, permeabilizing potency decreased (higher concentration required for equivalent results) and even slowed down (longer time needed) from **2** (-C_12_H_25_) through **6** (-C_10_H_21_) to **5** (-C_8_H_17_). The antifungal mechanism of action of the tested drugs evidently includes the incorporation of their molecules into the lipid bilayer, in which the hydrophobic alkyl chains are situated along the non-polar hydrocarbon chains of lipids and the positively charged pyridinium moiety settles in the polar environment near the plasma membrane surface. Longer tails most likely strengthen the interactions between styrylpyridines and lipids in the membrane. Since the studied compounds only contain one saturated aliphatic chain compared to the double-tailed lipids, they probably modify membrane fluidity and destabilize lipid membranes like detergents. Therefore the toxicity of the agents is dependent on cell density, because it influences the drug/membrane lipid ratio, and thus both the drug and cell concentrations determine the results. It also explains the apparent differences between toxic concentrations found in the growth tests (Figure 2) and in the estimation of cell plasma-membrane integrity (Figures 3, 4), because the cell density (OD) was 200× lower at the beginning of the growth tests than in the other experiments.

Since we found that all the styrylpyridinium derivatives were transported from *C. glabrata* cells as substrates of its MDR pumps, they would probably not be suitable for a single-drug antifungal therapy, but they may find their applications in combined therapy together with other antifungal drugs, which are often also substrates of MDR pumps. Styrylpyridinium compounds could increase the concentration of the given drug above the effective threshold due to the competition of two drugs for the binding sites of MDR transporters.

### Synergistic Effect of Styrylpyridinium Compounds With Antifungal Drugs

**FIGURE 5:**
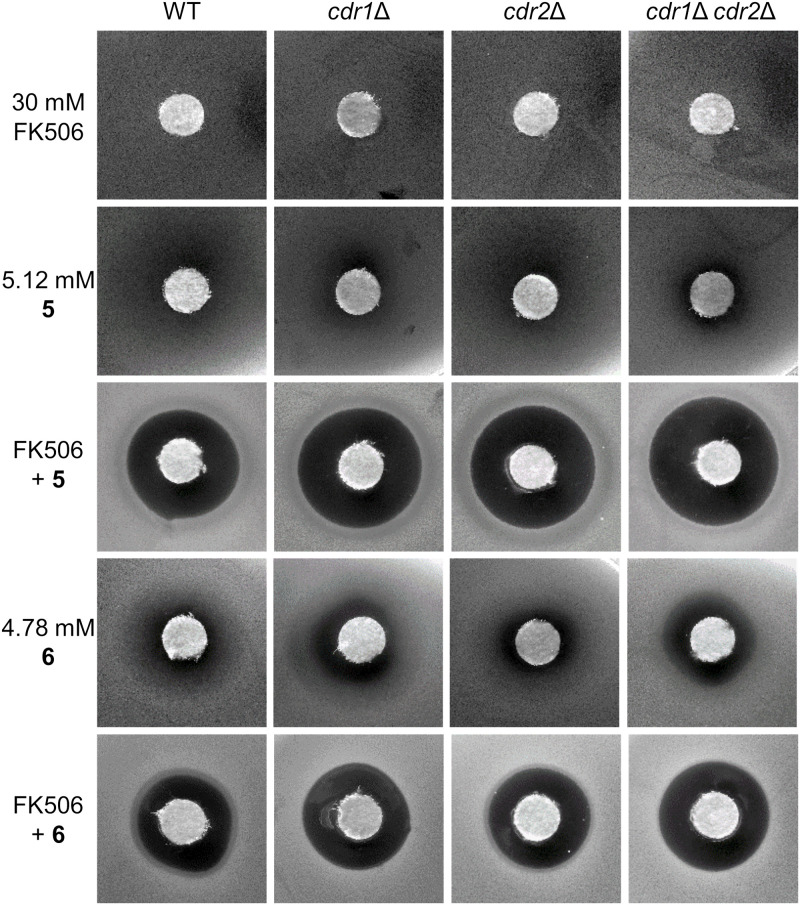
Growth inhibition zones for *C. glabrata* wild type and its mutants lacking Cdr1 and/or Cdr2 pumps exposed to FK506 or styrylpyridinium derivatives **5** and **6** at indicated concentrations and their combinations.

**FIGURE 6:**
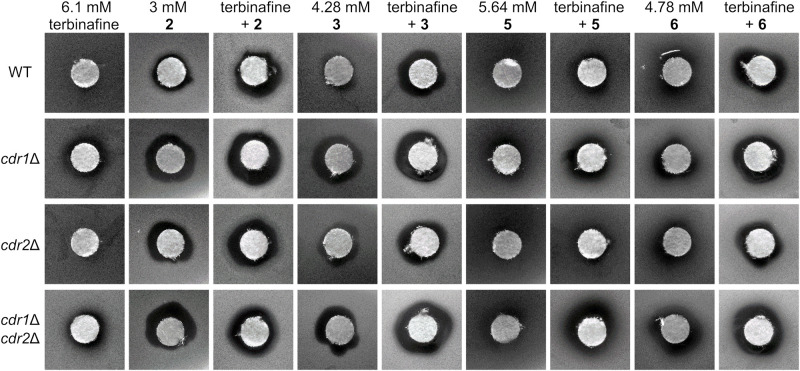
Growth inhibition zones for *C. glabrata* wild type and its mutants lacking Cdr1 and/or Cdr2 pumps exposed to terbinafine and styrylpyridinium derivatives **2**, **3**, **5**, and **6** at indicated concentrations and their combinations.

Combined therapy has become an important alternative for treating invasive fungal infections, leading to an improved efficacy of conventional antifungal drugs (such as azoles, amphotericin B or echinocandins) and at the same time reducing their side effects by using lower therapeutic doses. Several studies have shown a synergistic effect of azoles in combination with calcineurin inhibitors FK506 (tacrolimus) and cyclosporine A or other drugs (Mathé and Van Dijck, 2013; Denardi et al., 2015; Shrestha et al., 2015; Park et al., 2019). We performed disk diffusion tests to evaluate a possible synergistic effect of styrylpyridinium compounds **1**–**8** with FK506, terbinafine, fluconazole and ketoconazole against the four *C. glabrata* strains with and without Cdr pumps. Unfortunately, we did not observe any significant synergism between styrylpyridines and fluconazole or ketoconazole (data not shown), but we found strong synergism of some of the tested compounds in combination with FK506 (Figure 5) and terbinafine (Figure 6).

FK506 is a well-known and widely used immunosuppressant, which inhibits calcineurin phosphatase and prevents the T-cell activation and proliferation involved in transplant rejection. The antifungal action of FK506 is very similar to its immunosuppression mechanism, since the core components of the Ca^2+^-calcineurin signaling pathway, such as calmodulin and calcineurin, are conserved in eukaryotes, although the functions of these pathways differ between organisms (Cruz et al., 2002; Denardi et al., 2015; Park et al., 2019). In pathogenic fungi, calcineurin controls essential virulence pathways, such as the ability to grow at human body temperature, morphogenesis (hyphal or pseudohyphal growth), drug tolerance/resistance, and cell wall integrity (Park et al., 2019). The importance of the calcineurin pathway for fungal virulence makes its inhibitor FK506 a promising antifungal drug. Moreover, FK506 is able to act synergistically with azoles against *C. albicans* and *C. glabrata* (Cruz et al., 2002; Denardi et al., 2015).

In our case, when FK506 was applied alone at a 30 mM concentration on paper disks, it did not form any growth inhibition zones in the four *C. glabrata* strains used, but we observed a pronounced effect when it was combined with compounds **5** or **6** (Figure 5). Alone, compound **5** was only able to form bland zones (slightly decreased yeast density in diffusion areas around disks, which was the most prominent with the double mutant), however its combination with FK506 gave rise to large bright zones in all *C. glabrata* strains (Figure 5). Compound **6** gave similar results. Although alone this drug created wider and clearer zones than compound **5**, together with FK506 it made slightly smaller zones compared to compound **5**. Nevertheless, compound **6** alone achieved the clearest inhibitory effect against the double mutant, and FK506 markedly strengthened the fungicidal efficiency of this drug on all the tested strains (Figure 5).

While FK506 together with styrylpyridinium compounds **5** and **6** demonstrated excellent antifungal properties, these specific combinations may have limited applicability in human medicine because of host cross-reactivity resulting from immunosuppression caused by FK506. This restriction can be overcome with non-immunosuppressive FK506 analogs which keep their antifungal potency, such as L-685,818 and 9-deoxo-31-O-demethyl-FK506 or other FK506 derivatives with modified functional groups that modulate their antifungal and immunosuppressive activities (Jung and Yoon, 2020). On the other hand, the combination of styrylpyridinium compounds and FK506 can be directly advantageous for one group of patients: organ and bone marrow transplant recipients already receive calcineurin inhibitors as part of their immunosuppressive regime to prevent graft rejection and antifungal drugs (azoles) to treat concomitant fungal infections (Cruz et al., 2002).

Further, we tested the synergism of eight styrylpyridinium compounds and terbinafine, which blocks the ergosterol biosynthetic pathway in an earlier step (Erg1) than azoles. The resulting ergosterol depletion and squalene accumulation affects membrane structure and function (Campoy and Adrio, 2017). We suppose that these changes in the lipid composition of *C. glabrata* membranes may enhance the killing effectivity of styrylpyridinium compounds, since some reports have already described an increased efficiency of terbinafine in combination with other antifungal drugs on *C. albicans* or *C. dubliniensis* (Barchiesi et al., 1998; Scheid et al., 2012).

In our tests, only derivatives **2**, **3**, **5**, and **6** exhibited a significant synergistic effect in combination with terbinafine (Figure 6). Terbinafine applied alone formed quite small but bright inhibition zones for the two strains lacking Cdr1, as it is a substrate of this MDR pump (Puri et al., 2011). All of the above-mentioned hydroxy- and dimethoxystyrylpyridinium derivatives inhibited the growth of deletion mutants more than the wild type. Zones observed with *cdr1*Δ and *cdr1*Δ *cdr2*Δ strains were more noticeable than those observed for the wild-type strain, again validating our previous conclusion that most of the tested compounds are eliminated from cells by MDR pumps, predominantly Cdr1. We observed larger growth inhibition zones formed in the presence of terbinafine combined with styrylpyridinium derivatives possessing longer aliphatic chains (-C_12_H_25_ of compounds **2** and **3**, -C_10_H_21_ of drug **6**) than only an octyl substituent (**5**). Styrylpyridinium derivatives with longer alkyl chains are probably more tightly anchored between the surrounding lipids in *C. glabrata* membranes, because this yeast incorporates almost identical proportions of lipids with C16 and C18 fatty acids, in contrast to other *Candida* species that predominantly contain C18 fatty acids in membrane lipids (Kodedová et al., 2019). Terbinafine treatment initiates a drop in C18 fatty acids content in favor of C16 and an increase in the saturation of fatty acids in *C. glabrata* (Kodedová et al., 2019), which could even strengthen the interaction between similarly long chains of styrylpyridinium compounds and membrane lipids.

### Spectral Characterization of Styrylpyridinium Compounds

**FIGURE 7:**
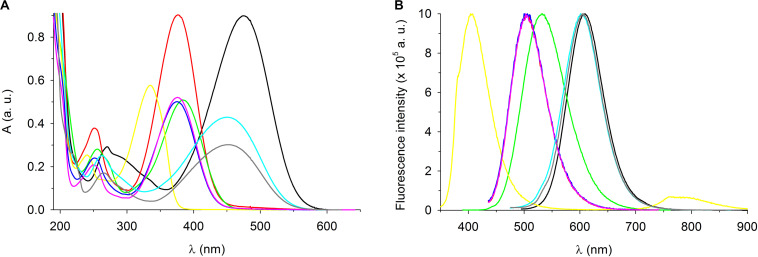
Spectroscopic characterization of styrylpyridinium derivatives. **(A)** Absorption and **(B)** fluorescence spectra of 20 μM styrylpyridinium derivatives in deionized water. Fluorescence spectra were normalized. Compound **1** (black line), **2** (red line), **3** (green line), **4** (yellow line), **5** (blue line), **6** (pink line), **7** (turquoise line), and **8** (gray line).

As was mentioned in the Introduction, some of the tested styrylpyridinium derivatives were originally synthesized as new fluorescence probes and their fluorescence spectra have already been published, e.g., compounds **5**, **7**, and **8** (Dubur et al., 1984; Krieg et al., 2007; Shiraishi et al., 2010). Some of the compounds used in this study have not yet been characterized in terms of how the length of their alkyl chain and specific substituents R1 and R2 on the aromatic ring (Figure 1 and Table 1) influence their spectroscopic properties. First, we measured the absorption spectra of 20 μM styrylpyridines in distilled water to find the optimal wavelengths for their excitation (Figure 7A). The absorption spectra of the whole group of drugs had the same main features, namely two major absorption peaks. The first, less intensive bands of all drugs, were found in the UV region. Their maxima were situated between 239 nm (compound **4**) and 271 nm (compound **1**). The second dominant absorption bands were shifted toward the visible spectrum, except for compound **4**. The entire absorption of compound **4** was clustered purely in the UV region (Figure 7A). Therefore, its solution was the only one that was colorless. Other drugs gave rise to yellow, orange or red solutions, depending on their concentrations. The maxima of these primary absorption bands (Figure 7A), summarized in Table 3, were used for the excitation of styrylpyridinium fluorescence. Normalized fluorescence spectra are shown in Figure 7B.

The shapes of all emission spectra were very similar, with full width at half maximum (FWHM) ranging from 69 nm (compound **4**) to 91 nm (compound **3**). The spectral half-width of each hydroxystyrylpyridinium derivative (**2**, **5**, and **6**) was identical (74 nm), indicating that this spectral parameter was evidently independent of the length of the aliphatic chain and instead defined by the R2 substituent (Table 1). This observation was also confirmed by four compounds (**1**–**4**) with the same dodecyl groups and different R2 substituents, whose emission bandwidths differed by up to 22 nm. Not only FWHM, but also the position of the maximum (both absorption and emission) was predominantly determined by the R2 substituents on aromatic rings, as documented in Figure 7 and Table 3. All hydroxystyrylpyridines emitted maximum fluorescence at 504–505 nm, therefore their emission spectra overlapped (Figure 7B), and also the differences between the positions of the absorption maxima were minimal (Table 3). Besides hydroxystyrylpyridines **2**, **5**, and **6**, the second group with overlapping fluorescence spectra in Figure 7B was composed of dimethylaminostyrylpyridines **7** and **8** and accompanied by diethylaminostyrylpyridine **1**. Whereas the fluorescence spectra of these three compounds were very close to each other, there were significant differences between diethylamino- (**1**) and dimethylaminostyrylpyridines (**7**, **8**) in the shape and position of their absorption peaks (Figure 7A and Table 3). Unlike the other dyes, the emission of compound **4** was localized to the shortest wavelengths of the spectrum (maximum λ_em_ = 405 nm), and we also registered emitted light by this drug in the near-infrared region.

The extraordinary large Stokes shifts of the tested styrylpyridines (Table 3) may be of significant benefit for their future biological applications. Common fluorophores such as fluorescein and rhodamine dyes exhibit small Stokes shifts (24–26 nm), which can reabsorb emitted photons leading to undesired background interference (Gao et al., 2017). The advantage of fluorescence dyes with large Stokes shifts (typically over 80 nm) is a minimization of cross-talk between the excitation source and fluorescence emission, meaning cellular imaging with a high signal-to-noise ratio (Gao et al., 2017). Excluding compound **4**, which has a Stokes shift of only 70 nm, the remaining derivatives more than fulfill the criterion of large Stokes shifts, which lengthen from hydroxy- (**2**, **6**, and **5** with 128, 129, and 130 nm) through diethylamino- (**1** with 135 nm) and dimethoxy- (**3** with 147 nm) to dimethylstyrylpiridines (**7** and **8** with 153 nm).

In summary, the small overlaps between the absorption and fluorescence spectra of all compounds (Figure 7) together with large Stokes shifts (Table 3) suggest good potential for their practical applications.

### Staining of *C. glabrata* Cells With Styrylpyridinium Derivatives

**FIGURE 8:**
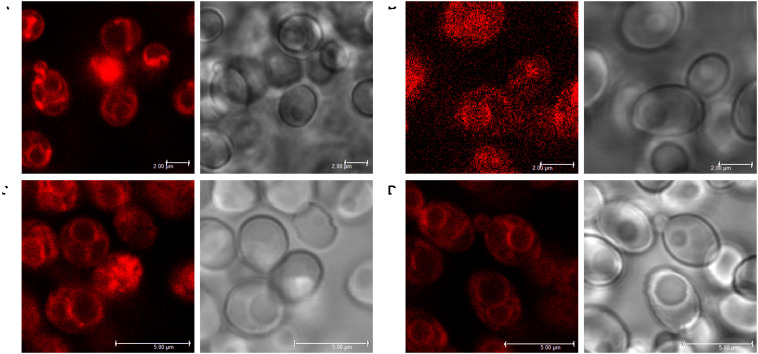
Staining of vacuolar membranes of *C. glabrata* wild-type cells with styrylpyridinium derivatives **1 (A)**, **3 (B)**, **7 (C)**, and **8 (D)**. Scale bar: 2 μm **(A,B)** or 5 μm **(C,D)**.

To confirm the biological applicability of the styrylpyridinium derivatives, we used confocal fluorescence microscopy to examine the cell staining and possible preferential localization of styrylpyridinium dyes within yeast cells. We chose a 405-nm laser line for the excitation of all compounds except for derivative **4**, which did not absorb light in this spectral region (its excitation was unfeasible under our conditions) and a 2 μM concentration, which did not seriously affect the membrane integrity of wild-type cells during short exposure (Figure 3) and corresponds to the concentration usually used for the commercial styrylpyridinium fluorescence dye FM4-64 (Miner et al., 2019). According to the distribution of fluorescence in *C. glabrata* wild-type cells, we can divide the tested compounds into two groups. The first group, comprised of compounds **1** (Figure 8A), **3** (Figure 8B), **7** (Figure 8C), and **8** (Figure 8D), was able to stain yeast plasma and vacuolar membranes, which exhibited higher fluorescence intensity compared to the cytoplasm with only dispersed fluorescence. With these compounds, we could observe several distinct vacuoles in each yeast cell (Figure 8), which is typical for an exponentially growing yeast culture. All derivatives with this specific distribution contained diethylamino-, dimethylamino- or dimethoxy- substituents, which evidently played analogous roles in the interaction with membrane structures such as the dibutylamino- group of the commercial styrylpyridinium vacuolar marker FM4-64. It is widely believed that FM4-64 and FM1-43 enter the cell primarily via endocytic vesicles invaginated from the plasma membrane and they insert into the membrane lipid bilayer via their lipophilic tails with the pyridinium dicationic head anchored at the membrane surface (Bolte et al., 2004). We suppose that the tested styrylpyridinium derivatives **1**, **3**, **7**, and **8** share enough essential molecular features with commercial styrylpyridinium dyes and utilize a similar entrance mechanism via endocytosis. However further studies will be necessary to prove this assumption.

**FIGURE 9:**
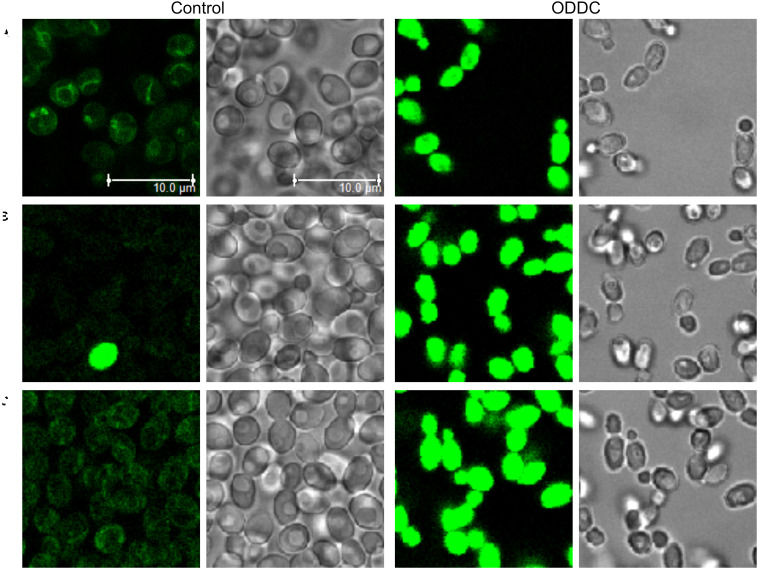
Increased accumulation of styrylpyridinium derivatives in dead *C. glabrata* wild-type cells. Images of living (control) and dead (permeabilized) cells (pretreated with ODDC) stained with styrylpyridinium derivatives **2 (A)**, **5 (B)**, and **6 (C)** were taken with identical settings of the confocal microscope for each drug, enabling relative comparison of fluorescence intensities. Scale bar: 10 μm for all pictures.

The second group of derivatives (**2**, **5**, and **6**) were not accumulated in any particular compartment of yeast cells (Figure 9). They stained whole yeast cells more homogenously. However, we discovered a striking difference between the fluorescence intensities of living and dead cells, e.g., one damaged cell visualized by compound **5** among living cells (Figure 9B). To confirm that these three compounds could be used as vital dyes, we treated *C. glabrata* wild-type cells with 3 μM octenidine dihydrochloride (ODDC) for 15 min, causing full permeabilization of their plasma membranes (Kodedová et al., 2019). These pretreated and washed cells were used as an example of killed cells for styrylpyridinium staining. We observed (Figure 9) a massive increase in the fluorescence intensity of dead cells stained by all three derivatives compared to the living (control) cells, which exhibited very low emission under identical settings of the confocal microscope for each drug, enabling a relative comparison of fluorescence intensities. Thus, depending on the structure of molecules, styrylpyridines can find their application as vital dyes (Figure 9) or vacuolar membrane probes (Figure 8).

We excited all styrylpyridinium compounds with a 405-nm laser, which is frequently used for the excitation of nuclear-specific DAPI or Hoechst dyes. But in contrast to these DNA binding probes, the emission of styrylpyridines is shifted toward longer wavelengths as indicated by their unusually large Stokes shifts. This fact makes them suitable candidates for the multi-color staining of cells. Moreover, compounds **1**, **7**, and **8** can be also very effectively excited at 440 or 473 nm. These λ_ex_ are often utilized for the excitation of the fluorescence proteins CFP and GFP. Compared to these cyan and green fluorescence proteins, the fluorescence of styrylpyridinium derivatives **1**, **7**, and **8** can be clearly detected in the red channel (>650 nm) and thus enables co-localization studies with CFP- or GFP-fused cell proteins. Simultaneously, our styrylpyridinium derivatives provide spectral alternatives to current tonoplast markers, because FM-dyes are excited by longer-wavelength lasers such as 488, 514, or 532 nm (Bolte et al., 2004).

Likewise, fluorescence microscopy clearly showed cell membranes to be the main target of styrylpyridinium compounds. Their antifungal activity, consisting of the permeabilization of the plasma membrane, was strongly dependent not only on exposure time, but also on achieving the threshold concentrations when they start to behave as detergents. Therefore the actual ratio of styrylpyridinium molecules per yeast cell seems to be crucial for the final effect, and a 2 μM concentration could be harmless during short-term exposures of abundant cells such as microscopy staining or the estimation of cell plasma-membrane integrity in contrast to the long-term exposure of the several hundred times smaller cell population in a growth test.

### Styrylpyridinium Derivatives as New Fluorescence Benchmark Substrates of *C. glabrata* MDR Pumps

We were interested in possible application of styrylpyridinium compounds as new fluorescence substrates of *C. glabrata* Cdr1 or Cdr2 pumps for monitoring efflux via these transporters. These drugs could not only enlarge a small group of fluorescence substrates suitable for study of *C. glabrata* MDR pumps, but also due to their completely different molecular structures from commonly used rhodamine 6G or diS-C_3_(3) probes, styrylpyridines can offer new information about substrate transport by *Cg*Cdr1 and *Cg*Cdr2. For this purpose, we select compound **8** as the least toxic derivative.

**FIGURE 10:**
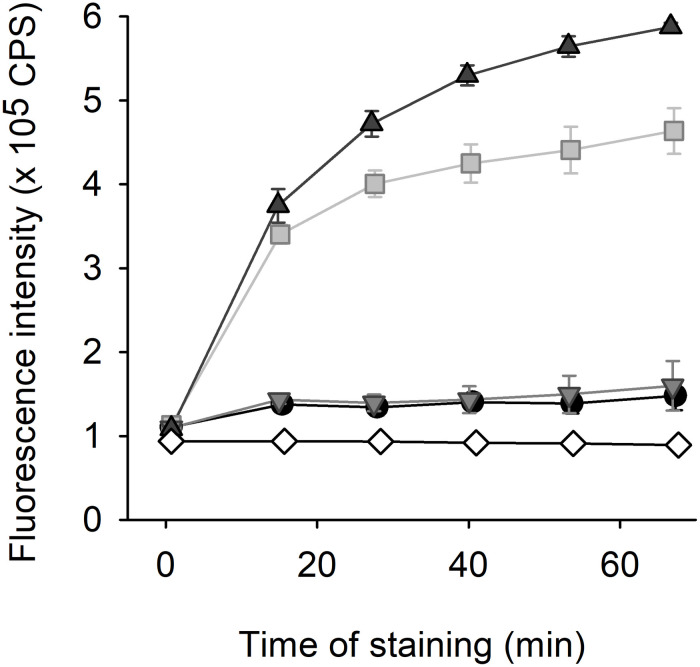
Fluorescence efflux assay with 2 μM styrylpyridinium compound **8**. The staining of *C. glabrata* wild-type (black circles), *cdr1*Δ (gray squares), *cdr2*Δ (light gray triangles), and *cdr1*Δ *cdr2*Δ (dark gray triangles) cells in comparison with staining of CP buffer without yeast cells (white diamonds).

Compound **8** responded to changes in its environment by altering its spectral characteristics. Maximum of its fluorescence emission measured in yeast cell suspension was shifted (in the range of several nanometers) to shorter wavelengths in comparison with the position in CP buffer or distilled water (data not shown), simultaneously, the intensity of fluorescence emission increased more considerably. Thus, we chose this spectral parameter for design of fluorescence efflux assay of compound **8** (Figure 10). The compound **8** exhibited a time- and strain-dependent accumulation in yeast cells accompanied by an increase in emission intensity. Strains lacking Cdr1 pump accumulated several times higher amount of the dye compared to the wild type. The difference between staining curves of *cdr1*Δ and *cdr1*Δ *cdr2*Δ strains suggested that also Cdr2 pump was able to eliminate compound **8** from *C. glabrata* cells. The negligible difference between wild-type and *cdr2*Δ cells was probably given by dominant role of *CDR1* due to its overexpression in these strains (unlike basal level of *CDR2*).

It was the first layout of the transport assay with styrylpyridinium compound (Figure 10) and several parameters would need to be optimized for routine applications. Nevertheless, according to our opinion, compound **8** has the greatest potential to become a new benchmark fluorescence substrate for finding new inhibitors of *Cg*Cdr1 as the least toxic styrylpyridinium derivative. Although we obtained similar results with its close derivative, compound **7** (data not shown), its higher antifungal activity could be undesirable in this application (analogously other derivatives that possess high antifungal activity).

## Conclusion

In summary, styrylpyridinium compounds may find their applications as new antifungal drugs or fluorescence dyes in yeast research. Compounds **2** (bromide of (E)-N-dodecyl-γ-azastilbenol-4′) and **6** (N-decyl 4-(*p-*hydroxy styryl) pyridinium bromide) exhibited the strongest inhibition of *C. glabrata* growth, followed by derivatives **1**, **5**, **4**, **3**, and **7**. The most promising antifungal agent is probably derivative **6**, which was able to effectively permeabilize the plasma membrane of yeast cells on its own and also exhibited significant synergism together with FK506 or terbinafine. On the other hand, the low toxicity of some derivatives could be beneficial for their application as fluorescence dyes, minimizing their side effects on yeast morphology. The least toxic derivative **8** (N-hexyl 4′-dimethylaminostyryl pyridinium tosylate) could serve as a new vacuolar marker, as its spectroscopic properties differ from current tonoplast markers and enable multi-color staining due to its exceptionally large Stokes shift (153 nm). Moreover, this compound can diversify the range of fluorescence benchmark substrates of *C. glabrata* Cdr pumps. The styrylpyridinium derivative **5** (N-octyl *p*-hydroxystyrylpyridinium bromide) can be used as new fluorescence vital dye, as it accumulates rapidly in dead cells and has a low killing potential.

## Data Availability Statement

The raw data supporting the conclusions of this article will be made available by the authors, without undue reservation.

## Author Contributions

SV and MK performed the experiments and analyzed the data. MK contributed to the conception of the study and designed the experiments. RD and HS supervised the study. SV, MK, and HS wrote the manuscript. All authors read and approved the final manuscript.

## Conflict of Interest

The authors declare that the research was conducted in the absence of any commercial or financial relationships that could be construed as a potential conflict of interest.
